# Non-traumatic Acute Subdural Hematoma in a Patient With Scleroderma Complicated by Pulmonary Arterial Hypertension: A Case Report

**DOI:** 10.7759/cureus.38769

**Published:** 2023-05-09

**Authors:** Uyioghosa Evbayiro, Thomas Delmas, Tasnim Lat

**Affiliations:** 1 Pulmonary and Critical Care Medicine, Texas A&M University School of Medicine, Temple, USA; 2 Neurocritical Care, Baylor Scott & White Medical Center - Temple, Temple, USA; 3 Pulmonary and Critical Care Medicine, Baylor Scott & White Medical Center - Temple, Temple, USA

**Keywords:** epoprostenol, prostacyclin therapy, subdural hematoma, pulmonary arterial hypertension, scleroderma

## Abstract

Non-traumatic acute subdural hematoma (SDH) in patients with scleroderma is infrequently described in literature reviewing the neurologic disorders in scleroderma. We report a case of a patient with scleroderma complicated by severe pulmonary arterial hypertension (PAH), and a history of pulmonary embolism on warfarin who developed an SDH, requiring hemicraniectomy after initiating therapy with IV epoprostenol. The proposed mechanisms for SDH development and management strategy are discussed.

## Introduction

Scleroderma is a rare autoimmune inflammatory disorder characterized by vascular dysfunction and fibrosis of the skin and multiple internal organs [1]. Involvement of organs such as the skin, lungs, or kidneys can lead to the development of progressive skin fibrosis, pulmonary arterial hypertension (PAH), and scleroderma renal crisis (SRC), among other manifestations. Patients with scleroderma can also have variable presentations of neurologic disorders with limited available literature describing manifestations. A review study on neurologic complications in scleroderma patients reported a 1% to 40% occurrence of neurological abnormalities in these patients, the most frequent being headaches, epilepsy, and cognitive impairment [1]. Reports of intracranial hemorrhage, more specifically non-traumatic subdural hematoma (SDH) in scleroderma patients, is infrequently described in literature reviewing neurologic disorders in scleroderma. Several cases describe scleroderma patients presenting with subarachnoid hemorrhage due to intracranial aneurysms with the conjecture that the perivascular fibrosis and microvascular damage induced by the autoimmune response in scleroderma may drive the process of aneurysm formation [2]. Subsequent aneurysm rupture may then lead to intracranial hemorrhage. We report a case of a patient with scleroderma complicated by severe PAH who developed a non-traumatic SDH after initiating prostacyclin analogue therapy. The proposed mechanisms for SDH development and management strategy are discussed.

## Case presentation

A 64-year-old female with a history of connective tissue disease-related PAH (CTD-PAH) due to scleroderma and pulmonary embolism (PE) treated with warfarin, presented to our pulmonary clinic to establish care for pulmonary hypertension after an initial workup and diagnosis made at an outside facility. She had a right heart catheterization (RHC) two years prior which revealed pulmonary vascular resistance (PVR) of 5.9 Woods units (WU) (≥3 WU is elevated), mean pulmonary artery pressure (mPAP) of 42 mmHg (normal range is 10 mmHg to 22 mmHg), and pulmonary capillary wedge pressure (PCWP) of 11 mmHg (normal range is 6 mmHg to 15 mmHg), consistent with precapillary PAH. Her PAH was previously managed with the oral phosphodiesterase-5-inhibitor sildenafil, and her scleroderma was managed with mycophenolate: an immunosuppressive agent. On presentation to our clinic, she reported limited exercise tolerance consistent with World Health Organization (WHO) functional class III-IV PAH symptoms. She underwent repeat RHC, revealing PVR of 16 WU, mPAP of 51 mmHg, and PCWP of 9 mmHg. Given her functional class and progression of severe PAH, she was admitted to the medical intensive care unit (MICU) for initiation of therapy with IV epoprostenol.

On admission to the MICU, her complete blood count (CBC) and comprehensive metabolic panel (CMP) were unremarkable, with an initial platelet count of 233 x 10*9/L and an international normalized ratio (INR) of 5.4. As she did not have active bleeding, warfarin was held without reversal, with plans to resume when INR levels became subtherapeutic due to her history of PE and the risk of in-situ thrombosis in severe PAH. She was started on IV epoprostenol at 1 ng/kg/min with up-titration every eight hours. While on IV epoprostenol, she had complaints of intermittent headache, nausea, and jaw pain that resolved with acetaminophen 650mg and norco (hydrocone-acetaminophen) 5/325 mg as needed.

**Figure 1:**
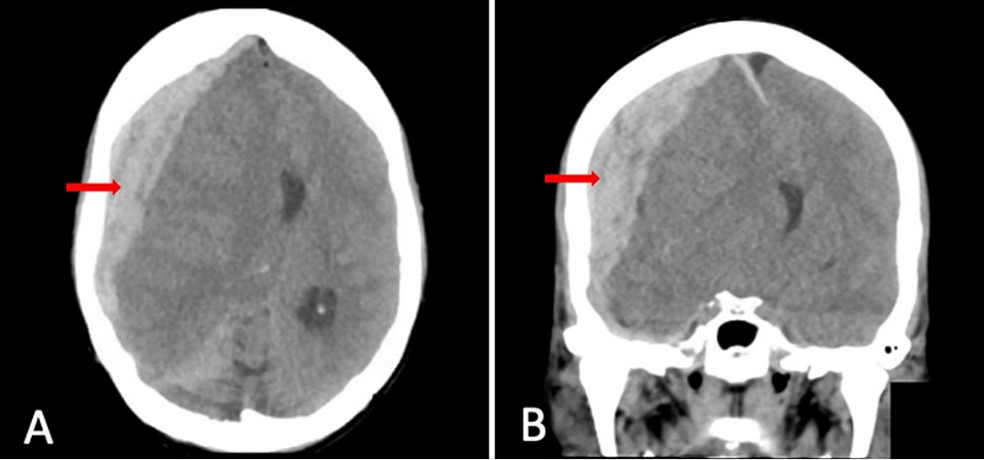
Non-contrast CT head revealing a 2 cm right-sided acute SDH (indicated by red arrows) with approximately 2.1 cm midline shift and subfalcine herniation SDH: Subdural hematoma, A: Axial view, B: Coronal view

On day four of admission, the patient was found slumped over in bed with no evidence of trauma and minimally responsive despite being awake, and conversant an hour earlier after receiving 5/325mg of norco. On exam, the MICU staff noted no eye-opening, no verbal response, and withdrawal to pain in the left upper and lower extremities, withdrawal to pain in the right lower extremity, and posturing in the right upper extremity consistent with a Glasgow coma score (GCS) of 6. She was given a 0.4 mg/ml injection of naloxone for possible norco-induced overdose with no improvement in neurologic status. Stat non-contrast CT head revealed a 2 cm, right-sided acute SDH with approximately 2.1 cm midline shift with subfalcine herniation (Figure 1). She was emergently intubated for airway protection, with concern for increased intracranial pressure with cerebral herniation. Subsequent evaluation by neurosurgery following the reversal of paralytics used for intubation with sugammadex was notable for GCS of 3T due to no eye-opening, no verbal response as she was intubated, and extension in all four extremities in response to pain. Labs revealed hemoglobin of 11.9, platelet count of 245 x 10*9/L, and INR of 2.6, with thromboelastography (TEG) unremarkable for evidence of dysfunction in coagulation. Anticoagulation and IV epoprostenol were subsequently held. She was given prothrombin complex concentrate (PCC) and vitamin K to reverse warfarin. Repeat INR after several hours was 1.3. She was subsequently taken by neurosurgery to the OR for emergent hemicraniectomy and evacuation of the SDH.

**Figure 2:**
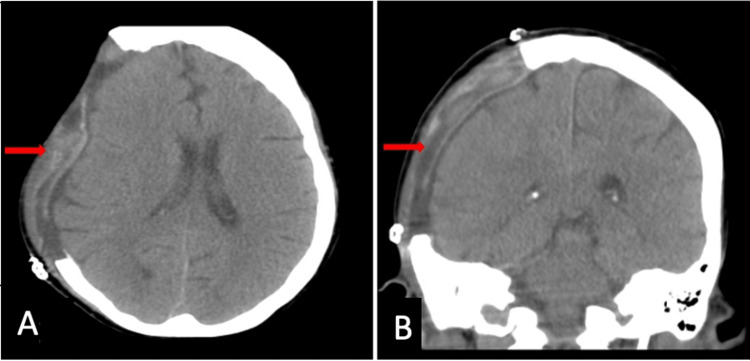
Non-contrast CT head post right hemicraniectomy (red arrows indicate the removal of bone flap and evacuated SDH) SDH: Subdural hematoma, A: Axial view, B: Coronal view

Same-day post-operative evaluation while intubated and sedated was notable for withdrawal to pain in all four extremities. Follow-up imaging with non-contrast CT head (Figure 2), and CT angiography of head and neck vessels did not provide evidence of vasculopathy that may have led to intracranial hemorrhage. The IV epoprostenol was not restarted due to concern for possible inhibition of platelet function and she was instead started on oral prostacyclin therapy with selexipag. She had a complicated ICU course requiring tracheostomy placement due to difficulty weaning off mechanical ventilation, and a percutaneous endoscopic gastrostomy (peg) tube was placed for nutrition. Toward the end of her ICU stay, her GCS improved to 14 as she was able to spontaneously open her eyes, speak with some confusion, and could follow commands in all four extremities. She was, however, only alert and oriented to self and had diffuse weakness in all four extremities. At the time of discharge, she was able to tolerate a tracheostomy collar in the morning and pressure support ventilation at night time. She was discharged with the tracheostomy and peg tube in place to a long-term acute care hospital for rehabilitation and follow-up with neurosurgery for the replacement of her bone flap.

## Discussion

Despite established reports of neurologic involvement in scleroderma patients, there are limited reports of non-traumatic SDH in these patients. Descriptions of brain imaging in scleroderma patients are significant for the presence of vasculopathy, white matter lesions, and brain calcifications. This may be indicative of cranial vessel involvement as a likely mechanism behind the development of neurologic disorders and may predispose to the fragility of bridging veins from which SDHs can form [3-5]. However, our patient did not have these reported abnormalities on brain and vessel imaging.

Our patient’s platelet count on presentation of her SDH was 245 x 10*9/L, making thrombocytopenia an unlikely cause for hemorrhage. While rare, there have been several cases of non-traumatic SDH described in PAH patients, mainly occurring in patients receiving epoprostenol while on anticoagulation with vitamin K antagonists independent of INR value [6-8]. Interestingly, CTD-PAH patients in the Henkens et al. study were also noted to have a significantly higher rate of major bleeding than other PAH sub-groups. The known inhibitory effect of prostacyclin analogues on platelet aggregation in addition to anticoagulant use was conjectured to be the primary mechanism driving increased rates of major bleeding compared to other PAH therapies and is likely a major risk factor for the development of non-traumatic SDH in our patient.

While our patient’s SDH required hemicraniectomy, less severe non-traumatic SDHs can also be managed conservatively with serial neurologic exams, and serial head imaging. Indications for neurosurgical intervention include hematoma size ≥10 mm, evidence of herniation, acute neurologic change, and failure of conservative management [9,10]. For patients with PAH, epoprostenol can be held if there is a concern for inhibition of platelet function but should only be done after consulting with a pulmonary hypertension specialist as case reports of surgically managed SDH in the setting of prostacyclin use have described potential rebound pulmonary hypertension leading to right-heart failure as a concern if PAH therapy is abruptly discontinued [8,11].

Thromboelastography may not be a reliable study to assess platelet function and determine whether platelet inhibition by epoprostenol is contributing to the development of SDH. It has been validated in assessing global hemostasis and guiding transfusion therapy in the setting of trauma or surgery. Platelet function tests such as light transmission aggregometry (LTA) and platelet function analyzer (PFA) could be more reliable studies as they assess platelet adhesion and aggregation which can be inhibited by prostacyclin analogues [12]. To our knowledge, this is one of a very limited number of non-traumatic SDH cases in patients with scleroderma and PAH with potential risk factors of concomitant anticoagulant and prostacyclin analogue therapy. Further research is warranted to delineate risk factors predisposing to non-traumatic SDH and the possibility of other forms of non-traumatic intracranial hemorrhage such as epidural hematomas, subarachnoid hemorrhage, and intraparenchymal hemorrhage in scleroderma patients. This will aid in the development of management strategies that are suitable to address each type of intracranial hemorrhage. 

## Conclusions

The reported presence of vessel abnormalities on head imaging and the spectrum of neurological symptoms in scleroderma patients may potentially serve as risk factors for the development of non-traumatic SDH in scleroderma. Likewise, concurrent prostacyclin therapy and anticoagulant use may be additional risk factors for the development of non-traumatic SDH in these patients. Caution should be exercised when initiating therapy with prostacyclins and anticoagulants for PAH, and lab studies that can accurately assess platelet function and aggregation should be used to help monitor bleeding risks. More studies are needed to quantify the risk of non-traumatic SDH and other forms of non-traumatic intracranial hemorrhage in patients with scleroderma. 

## References

[1] Amaral TN, Peres FA, Lapa AT, Marques-Neto JF, Appenzeller S (2013). Neurologic involvement in scleroderma: a systematic review. Semin Arthritis Rheum.

[2] Jabre R, Benomar A, Bojanowski MW (2020). Scleroderma's possible dual role in the pathophysiology of intracranial aneurysms: case report and literature review. World Neurosurg.

[3] Kandemirli SG, Bathla G (2021). Neuroimaging findings in rheumatologic disorders. J Neurol Sci.

[4] Kister I, Inglese M, Laxer RM, Herbert J (2008). Neurologic manifestations of localized scleroderma: a case report and literature review. Neurology.

[5] Terrier B, Charbonneau F, Touzé E (2009). Cerebral vasculopathy is associated with severe vascular manifestations in systemic sclerosis. J Rheumatol.

[6] Henkens IR, Hazenoot T, Boonstra A, Huisman MV, Vonk-Noordegraaf A (2013). Major bleeding with vitamin K antagonist anticoagulants in pulmonary hypertension. Eur Respir J.

[7] Louis L, Bair N, Banjac S, Dweik RA, Tonelli AR (2012). Subdural hematomas in pulmonary arterial hypertension patients treated with prostacyclin analogs. Pulm Circ.

[8] Rammo R, Robin A, John J, Pabaney A, Varelas P, Kole M (2017). Management of acute subdural hematoma in a patient with portopulmonary hypertension on prostanoid therapy. Surg Neurol Int.

[9] Fomchenko EI, Gilmore EJ, Matouk CC, Gerrard JL, Sheth KN (2018). Management of subdural hematomas: part II. Surgical management of subdural hematomas. Curr Treat Options Neurol.

[10] Gerard C, Busl KM (2014). Treatment of acute subdural hematoma. Curr Treat Options Neurol.

[11] LeVarge BL (2015). Prostanoid therapies in the management of pulmonary arterial hypertension. Ther Clin Risk Manag.

[12] Tyagi T, Jain K, Gu SX (2022). A guide to molecular and functional investigations of platelets to bridge basic and clinical sciences. Nat Cardiovasc Res.

